# Methods of international health technology assessment agencies for economic evaluations- a comparative analysis

**DOI:** 10.1186/1472-6963-13-371

**Published:** 2013-09-30

**Authors:** Tim Mathes, Esther Jacobs, Jana-Carina Morfeld, Dawid Pieper

**Affiliations:** 1 Institute for Research in Operative Medicine, Faculty of Health - School of Medicine, Witten/Herdecke University, Ostmerheimer Str. 200, Building 38, D - 51109 Cologne, Germany

## Abstract

**Background:**

The number of Health Technology Assessment (HTA) agencies increases. One component of HTAs are economic aspects. To incorporate economic aspects commonly economic evaluations are performed. A convergence of recommendations for methods of health economic evaluations between international HTA agencies would facilitate the adaption of results to different settings and avoid unnecessary expense. A first step in this direction is a detailed analysis of existing similarities and differences in recommendations to identify potential for harmonization. The objective is to provide an overview and comparison of the methodological recommendations of international HTA agencies for economic evaluations.

**Methods:**

The webpages of 127 international HTA agencies were searched for guidelines containing recommendations on methods for the preparation of economic evaluations. Additionally, the HTA agencies were requested information on methods for economic evaluations. Recommendations of the included guidelines were extracted in standardized tables according to 13 methodological aspects. All process steps were performed independently by two reviewers.

**Results:**

Finally 25 publications of 14 HTA agencies were included in the analysis. Methods for economic evaluations vary widely. The greatest accordance could be found for the type of analysis and comparator. Cost-utility-analyses or cost-effectiveness-analyses are recommended. The comparator should continuously be usual care. Again the greatest differences were shown in the recommendations on the measurement/sources of effects, discounting and in the analysis of sensitivity. The main difference regarding effects is the focus either on efficacy or effectiveness. Recommended discounting rates range from 1.5% - 5% for effects and 3% - 5% for costs whereby it is mostly recommended to use the same rate for costs and effects. With respect to the analysis of sensitivity the main difference is that oftentimes the probabilistic or deterministic approach is recommended exclusively. Methods for modeling are only described vaguely and mainly with the rational that the “appropriate model” depends on the decision problem. Considering all other aspects a comparison is challenging as recommendations vary regarding detailedness and addressed issues.

**Conclusion:**

There is a considerable unexplainable variance in recommendations. Further effort is needed to harmonize methods for preparing economic evaluations.

## Background

“Health technology assessment (HTA) is a method of evidence synthesis” [1]. The number of agencies conducting HTA is increasing worldwide [2]. Economic factors are an integral part of HTA [1]. Incorporating them into decision making has become more important in recent years as new drugs enter the market, showing no or only marginal additional benefits [3]. When incorporating economics into HTA reports, primary economic evaluations or systematic reviews of economic evaluations are applied. The advantage of a primary evaluation is that it can be fitted optimally to the underlying problem and context. Economic evaluations are well-established in HTA [4]. The main aim of HTA agencies is to inform decision makers about policies [2]. The results of economic evaluations are commonly used in HTA to support reimbursement or coverage decisions. Thus, on one hand, a minimum standardization of the evaluation process is essential for transparent and comprehensible comparisons between different technologies and to ensure evaluations’ methodological quality. On the other hand, a complete standardization in all circumstances can be inappropriate because the methods should be flexible enough to be compatible with different problems in different contexts [5]. This trade-off requires a careful balance to produce valid decisions accepted by both society and other involved stakeholders. Although efforts to standardize economic evaluations were initiated in the 1990s [6], for some methodological issues, there is still no consensus regarding the “appropriate” method or the optimal grade of standardization in scientific society. Especially in decision modeling, several different approaches can potentially be applied [7]. Prior research reveals that recommendations for modeling according to scientific guidelines are partly in conflict [8]. Without any doubt, methods of economic evaluation should be adapted to health care systems and settings and specific requirements in different countries [9]. However, in addition to these justifiable differences, there are also methodological aspects that are independent of the setting and health care system for which an evaluation is prepared. An agreement regarding use these methods among international HTA agencies, in conjunction with transparent reporting, would facilitate interpretation and adaption of results to different settings. The same health technology (HT) is often assessed multiple times by different HTA agencies. Thus, a simple adaption could help to avoid unnecessary duplicate work and expense. How widely current guideline recommendations of public HTA agencies differ between countries and, consequently, the domain requiring harmonization, is unknown.

The objective of this paper is to provide a comprehensive overview and comparison of recommendations of international HTA agencies regarding methods of preparing economic evaluations in an attempt to identify both differences and potential for harmonization.

## Methods

We systematically searched webpages of international HTA agencies and institutes involved in HTA for handbooks, manuals, and guidelines containing recommendations on the preparation of economic evaluations. The HTA agencies were identified through member lists of the International Network of Agencies for Health Technology Assessment (INAHTA), Health Technology Assessment International (HTAi) non-for-profit organizations and the European Network for Health Technology Assessment (EUnetHTA). In total, the webpages of 127 agencies were searched independently by two reviewers in September 2012. Furthermore, to capture unpublished manuals, which were not identified by the webpage search, all agencies were contacted by e-mail in the first week of October 2012. Contact details were obtained from the webpages of INAHTA, HTAi and EUnetHTA. Replies were accepted through December 31^st^ 2012.

Inclusion criteria were as follows:

1. publications written either in English or German

2. methods manual, guidelines, etc. describing methods of preparation of HTA reports

3. methods manual, guidelines, etc. containing specific recommendations on methods of preparation of health economic evaluations.

No exclusion criteria were applied. Publication date was not restricted. If different versions of the same document were available, only the most current document was included. Two reviewers independently screened the identified publications for relevance according to the inclusion criteria. Discordant screening results were resolved by discussion until consensus was reached, or through consultation with an unbiased third party.

To synthesize the data, standardized tables were prepared a priori containing the headings: purpose of economic evaluation; type of evaluated technologies; types/choice of evaluation/outcomes; choice of alternatives; time horizon; perspective; costs; measurement/sources of resource use; valuation of resource use; measurement/valuation of outcomes; sources of outcomes; discounting per year; modeling; type/parameters for analysis of sensitivity/uncertainty; and equity aspects. These were chosen for their relevance to the preparation of economic evaluations and presentation of results. The data were extracted by one reviewer and checked by a second reviewer to assure quality. Discrepancies were resolved by discussion until consensus was reached, or through consultation with an independent third party. Information in these tables, unless noted otherwise, refers to the base case scenario/reference case of the economic evaluation, except for the item analysis of sensitivity/uncertainty. With respect to the analysis of sensitivity, the extraction refers to parameter and structural uncertainty. All recommendations were standardized when extracted. General (scientific) descriptions of methods were not extracted. When extracting statements that were not clearly denoted as recommendations, two reviewers decided whether they could be interpreted as recommendations. It was assumed that, as with clinical guidelines, recommendations were intentionally formulated by the HTA agencies to showcase the strength of each recommendation. Therefore, in the tables, the wording of the recommendations was extracted from the documents as exactly as practicable while avoiding interpretation bias. In contrast, to summarize the results in the main text, it was necessary to disregard different formulations and expressions (e.g., usual care vs. standard care, payer perspective vs. funder perspective).

## Results

### Search results

Overall, 63 potentially relevant publications (in English or German) were identified. Following title and abstract screening, 36 publications were excluded. Seven of the excluded publications were not manuals (or process guidelines); 29 of them did not contain recommendations for economic evaluations. Therefore, 27 manuals fulfilled all inclusion criteria. The additional inquiries via e-mail revealed no further relevant manuals. The response rate was 28%.

Two agencies (SingHealth and Dental and Pharmaceutical Benefits Agency) do not publish their own sources but refer to other scientific manuals instead, which are used as a basis for their evaluations [10,11]. Therefore, these publications were excluded because they did not contain any recommendations. Some agencies provided multiple manuals, which addressed specific methodological issues of preparing economic evaluations (e.g., modeling). In the end, 25 manuals provided by 14 HTA agencies [7,12-34] were included. The agencies are listed below:

1. Agency for Health Technology Assessment in Poland (AHTApol/Poland) [20]

2. Canadian Agency for Drugs and Technologies in Health (CADTH/Canada) [26]

3. Dutch Health Care Insurance Board (CVZ/Netherlands) [18,23]

4. Danish Centre for Health Technology Assessment (DACEHTA/Denmark) [27]

5. Gesundheit Oesterreich GmbH (GOEG/Austria) [7]

6. Health Information and Quality Authority (HIQA/Ireland) [25]

7. National Authority of Medicines and Health Products (INFARMED/Portugal) [21]

8. Institute for Quality and Efficiency in Health Care (IQWIG/Germany) [13,28,31-33]

9. Belgian Federal Health Care Knowledge Centre (KCE/Belgium) [22]

10. Medical Advisory Secretariat within the Ontario Ministry of Health and Long-Term Care Health Strategies Division (MAS/Canada) [29]

11. Medical Services Advisory Committee (MASC/Australia) [17,24]

12. National Institute for Clinical Excellence (NICE/UK) [14,19]

13. Pharmaceutical Benefits Advisor Committee (PBAC, Australia) [35]

14. Pharmaceutical Management Agency of New Zealand (PHARMAC/New Zealand) [15,16,30].

### Analysis of methods of economic evaluations

The purpose of the economic evaluations, the evaluated technologies and recommendations for evaluation of the individual HTA agencies are presented in Additional file 1: Table S1.

### Purpose of evaluation

All considered HTA agencies were completely or partly publically funded. With the exception of the manuals from three HTA agencies [7,20,27], the purpose of the manuals is to specify methods for reports used to support reimbursement decisions [12,17,19,21-23,25,26,29,30],[35].

### Evaluated technologies

Seven agencies evaluated all types of HT or made no specification of the HT selected for economic evaluation [7,12,20,24-27]. Five HTA agencies evaluated exclusively drugs [21,22,29,30,35]. CVZ states that its guidelines are intended for designing and conducting pharmacoeconomic research, but that they are also applicable to economic evaluations in general [22]. NICE evaluates pharmaceuticals, medical devices, diagnostic techniques, surgical procedures, other therapeutic technologies and health promotion activities [19].

### Types/choice of evaluation/outcomes

All HTA agencies recommend a cost-utility analysis (CUA) or a cost-effectiveness analysis (CEA) as a primary type of analysis for economic evaluations; a CUA is mostly preferred or exclusively accepted. In most agencies, different types of analyses can be performed in parallel. PHARMAC is the only agency relying exclusively on CUA [30]. The IQWiG specifies the type of clinical outcomes, mortality, morbidity and validated surrogates as the outcomes required, and not the type of analysis [12].

### Choice of alternatives

Usual care is required by the primary comparator of 11 agencies [17,19,20,22,23,25-27,30,35]. The least expensive treatment alternative, recommended/guideline care or all relevant alternatives are often recommended for further analysis. The IQWiG recommends the continuous comparison of all relevant alternatives [12]. MAS recommends using the least expensive alternative treatment alternative [29].

### Time horizon

Time horizon is consistently required to be either long enough to capture all relevant costs and benefits or dependent on the duration of disease or lifetime period [16-18,20,22,23,25,26,28,32],[33,35]. MAS recommends choosing a time horizon depending on data from which estimates can be derived [29].

### Perspective

Six agencies recommend the societal perspective for the analysis [17,21,23,27,29,35] and four recommend [20,25,26,30] the funder perspective. NICE uses the funder and personal social services to estimate costs and KCE uses the funder and private expenditures. Regarding outcomes, both apply the societal perspective [19,22]. IQWIG requires applying the perspective of the socially insured [12].

### Costs

The perspectives used for evaluations vary and consequently the agencies include different costs depending on the type of analysis employed. Beyond this variation, the costs included differ further between the agencies. The only consistency throughout all agencies is the inclusion of direct medical costs. Many agencies also include direct costs outside the health care system [7,12,17,21,22,25,26,29],[30]. Additionally, indirect costs included in the societal perspective differ. Some agencies include, for example, only the productivity loss [33] while others also include the time cost of families [11]. Intangible costs are not included by any agency.

### Measurement/sources of resource use/costs

Most agencies recommend measuring resource use in natural units [10,11,16,24,25,27,28,33],[35]. If the costing approach is addressed, micro-costing is preferred [7,20,26]. It is sometimes recommended to identify resource use systematically [19,25]. Recommended sources range from the collection of medical literature (e.g., randomized controlled trials, guidelines) across administrative data to expert opinions. There is often no one certain source recommended; however, different possible sources are suggested for consultation [20,25]. Some agencies mention that national data should be used [19,21]. To address productivity loss, both the friction cost approach and the human capital approach are suggested [12,23,35].

### Valuation of resource use

Many agencies require that costs have to reflect opportunity costs [12,17,22,27,35]. For valuation of market prices, list prices and administrative data regarding hospital costs (e.g., DRGs) are mostly recommended [10-13,16,17,25,28,31,35]. The valuation of labor is only addressed by one agency, which requires the use of consolidated salary scales for calculation [25].

### Measurement/valuation of outcomes

The measurement/valuation of outcomes is predominantly described in cost-utility analysis. With respect to the assessment of health-related quality of life, most agencies recommend the use of generic instruments [17,19,21,22,25,27,29,30],[35]. Specific instruments, if any, are only recommended in combination with generic instruments [21,22]. Direct methods are recommended more often than indirect methods. Three agencies recommend using measures from patients affected by a disease. Only CADTH recommends using a representative sample of the public [26]. Outcomes for CEA are addressed in two agencies [21,22]. KCE recommends the use of life expectancy for chronic conditions [22] but PHARMAC states that outcomes should relate to treatment and life duration. IQWiG uses mortality, morbidity and validated surrogates as outcomes for analysis. For monetary outcomes, three agencies recommend willingness-to-pay [21,26,29]. DACEHTA recommends not using willingness-to-pay in isolation [27].

### Sources for outcomes

Clinical trials (efficacy) or meta-analyses of clinical trials are the preferred sources of outcome data in six agencies [7,19,21,25,30,35]. In contrast, AHTApol prefers to use data from observational studies [20]. Five agencies recommend using both [12,22,23,26,27]. A systematic literature search to locate studies is required by seven agencies [7,12,17,19,25,29,35].

### Discounting

Nine agencies recommend using the same discount rate for both cost and health benefits [7,12,19,21,24-26,30,35]. Three agencies require higher discount rates for costs than for benefits [20,22,23]. The highest difference between rates is 3.5 percentage points [23]. The annual discount rate ranges from 3 to 5% for costs and from 1.5 to 5% for benefits.

### Modeling

With regard to modeling, none of the agencies specifies the model type a priori. In addition to the cycle length correction of Markov-models [20,25,30], there are no precise recommendations for modeling but instead general descriptions. Three agencies recommend a systematic review to determine model input parameters [7,20,26].

### Analysis of sensitivity/uncertainty

Two agencies primarily recommend a deterministic sensitivity analysis (DSA) but consider a probabilistic sensitivity analysis (PSA), as well [26,32]. Inversely, one agency recommends PSA and additionally considers DSA [22]. Six agencies address the type of sensitivity analysis but generally consider both types of sensitivity analyses [20], depending on the assessed variables [19,23,25] or on the number of varied parameters [7,30]. The variation of the discount rate is required by ten agencies [7,20,21,24-27,30,35,36]. The ranges of costs and benefits fall mostly between 0% and 5%, but can reach up to 10% [7]. Structural uncertainty is addressed by nine HTA agencies [7,19,22,25-27,30,32,35]. All agencies recommend testing for structural uncertainty. Eight HTA agencies recommend the use of a sensitivity [7,22,25-27,30,32,35] or scenario analysis [19,25,37] for this purpose [7,22,26,27,30,32,35].

### Equity aspects

Equity is considered by five agencies [7,19,25,26,35]. Two agencies recommend considering relevant subgroups [7,26]. Furthermore, CADTH recommends identifying equity-relevant characteristics and providing distribution and cost-effectiveness information for these subgroups [26]. CADTH, HIAQ and NICE require the use of the same equity weights for QALYs/outcomes [7,19,25,26]. PBAC recommends testing equity assumptions via sensitivity analysis [35].

### Presentation of results

The presentation of results is described by twelve HTA agencies [7,12,17,19,21,22,25-27,29],[30,35]. Most agencies recommend presenting the results in disaggregated form or both disaggregated and aggregated forms [17,19,22,25,26,35] and presenting incremental-cost-effectiveness ratios [16,17,19,22,26,27,30,35]. Often, agencies recommended presenting results in tables or graphically [25,26,37], especially when presenting results of uncertainty analysis [17,30].

## Discussion

Many international HTA agencies require health economic evaluations [12,17,19,21-23,25,26,29,30],[35]. In the majority of agencies, economic evaluations are carried out to support reimbursement decisions [17,19,21,25,26,29,30,35]. The results of the economic evaluation are either used to decide whether the HT is generally covered by the funder or for price negotiations with the industry (or both). They are more often used for general coverage decisions (e.g., is an HT covered?) in state-funded health care systems because their budgets can be regarded as mainly fixed. Some HTA agencies evaluate exclusively pharmaceuticals. The focus on pharmaceuticals is probably because drugs are the HT representing the highest proportion of total health care costs and the methods for the economic evaluation of drugs (pharmacoeconomics) are well established.

Most agencies in state-funded health care systems recommend or require exclusively cost-utility analyses in the base-case scenario [19,21,25,26,30]. This is probably because a cost-utility analysis enables comparison between different indications and types of HT; especially in state-funded health care systems, reimbursement decisions are commonly made encompassing all indications. Furthermore, CUA enables a higher level of standardization because the same denominator is used for all types of HT and the methods to determine it can be better standardized. In contrast, social insurance systems mostly prefer cost-utility analyses or cost-effectiveness analyses [12,22,23]. A cost-effectiveness analysis can be applied because the HT is commonly only compared with one indication. Thus, indication-specific outcomes, especially patient reported outcomes (e.g., specific quality-of-life measurements) or proofed surrogates (e.g., blood pressure), can be used for comparison. However, the consequences of full standardization of both methods for outcomes in cost-effectiveness analysis and the definition of additional benefit or a fixed cost-effectiveness threshold are difficult to predict because there are too many outcomes that can potentially be used. The decision regarding a HT is therefore complicated and transparent. The methods can be further harmonized because, apart from the methodological difficulties of cost-utility analysis (e.g., measuring quality of life, multiplication of quality of life and live years), there is no reason to prefer cost-effectiveness analysis. This is especially true considering that HTA agencies give no concrete descriptions of cases where a cost-effectiveness-analysis is accepted or recommended. Furthermore, methodological problems (e.g., measurement) can also be relevant in cost-effectiveness analysis. A cost-benefit analysis is not accepted in the base case scenario by almost all HTA agencies. This indicates that valuing health outcomes in monetary units (e.g., through the willingness-to-pay or willingness-to-accept approach) is not considered to be best option for economic evaluation of HTs. The main reasons that cost-benefit analysis is not recommended are likely ethical concerns about the low acceptance of valuing health in monetary units in many societies and, consequently, low acceptance of decisions. Furthermore, there are methodological difficulties in valuing health in a monetary fashion (e.g., a willingness-to-pay stated preference depends on income) [38].

Almost all HTA agencies recommend one main comparator. This comparator is mostly usual care (e.g., standard care) or existing practice. The different formulations were probably chosen very consciously by the HTA agencies although each basically means the same thing. Nevertheless, in some cases, slightly different formulations can result in different comparators for the same HT or imply different degrees of flexibility for the choice. For example, usual care can mean “all HTs that are usually applied in practice”. Standard care is an accepted treatment (e.g., described in clinical guidelines) but not necessarily the most used. Only the IQWIG requires comparisons with all these alternatives; this is necessary to estimate a cost-effectiveness frontier for multiple interventions [39].

Many HTA agencies recommend choosing a time horizon depending on duration of disease, but this is often only useful for a societal perspective. Most of the other HTA agencies recommend choosing a time horizon depending on relevant costs and benefits; this is vague, but at least implies recognition of the importance of perspective of analysis. In addition to a lifetime period for chronic conditions, there are no other exact specifications of time horizons for analysis. For example, there is no recommendation for vaccinations that could have longer (economic) effect than a disease, or even a lifetime period because of herd immunity. Thus, the scientific literature suggests that the time horizon of an economic evaluation should be long enough to capture all observable health outcomes and effects caused by a technology [10] independent of disease duration. The majority of HTA agencies in state-funded health systems recommend using the perspective of the funder or all national social services [19,20,25,26,30]. For outcomes, this implies mostly a societal perspective because health care systems in these countries embrace the entire society. Additionally, regarding cost, this perspective is relatively broad: social services mostly have substantial coverage. In social insurance systems, the societal, social insured or the funder perspective is recommended. Principally, the societal perspective is adopted. The state-funded health care systems are funded by taxes paid by the entire population. Consequently, the proper perspective is all who pay, i.e., the whole society. In social insurance systems, a societal perspective is desirable because the main proportion of the societies is covered and the systems are publically funded. Furthermore, to subtract out the corresponding proportion of persons not covered is practically impossible, especially for health effects concerning a population (e.g., infectious diseases). None of the HTA agencies suggest that the perspective of analysis can depend on the indication so that costs or outcomes do not differ between different perspectives (e.g., acute conditions). If all HTA agencies used the societal perspective, the adaptation of economic evaluations to different countries would become easier: all arising costs of the disease would be considered independently of who bears the costs.

Recommended costs that should be included vary depending on the chosen perspective. However, substantial unjustifiable differences exist. Some agencies include only direct medical costs, while others also include non-medical direct costs, such as patient and family time. Often, payments that must be made by the patient are explicitly mentioned. In the societal perspective, either all indirect costs or only productivity losses are recommended for inclusion. All HTA agencies should consider all relevant changes in cost caused by HT, irrespective of cost type. Focusing on certain cost types may lead to improper decisions because HTs can have different effects on different cost types. Furthermore, the restriction to a certain cost type can result in missing costs that would otherwise have had a strong impact on the evaluation, especially in a societal perspective. All differences caused by the compared technologies are relevant. This would also facilitate the transferability of results.

Measuring resource use in natural units seems to be generally accepted. This simplifies the transferability of results because it can be presumed that differences in cost of HTs are primarily attributable to differences in prices, not resource use [40]. The measurement in natural units allows adaptation: only the used resources have to be newly priced in country specific unit prices. A completely new measurement of costs is not necessary. None of the HTA agencies included intangible costs in the denominator, reflecting that this cost type is seldom used because of methodological difficulties (the same cost influences the numerator as well as the denominator in the analysis). Especially in cost-utility analysis, such cost effects are implicitly integrated into the denominator. Unfortunately, a more detailed comparison of included costs is difficult because recommendations for the measurement and sources of costs vary in detail and addressed issues. One reason is perhaps that sources of resource use and costs depend on national circumstances, such as the availability of databases containing national data. However, there seems to be a tendency to identify resource use systematically, e.g., by a systematic literature search. Regarding the valuation of costs, most agencies recommend the use of opportunity costs. Market prices, as well as list prices, and administrative data are suggested for approximation. Some recommendations within one HTA agency are in conflict with the demand to measure resource use in natural units respectively a micro-costing approach. Recommendations are heterogeneous among the HTA agencies. However, the mechanism of price formation depends upon both reimbursement systems and HT. Thus, the variations seem mostly justifiable. A harmonization is not suggested because prices depend strongly on the national economy also, if market prices are exclusively used.

Because there are many possible outcomes that can be measured in natural units, it is logical that the measurement and the valuation of outcomes for health-related quality of life used to estimate QALYs are described. With few exceptions, generic direct measures taken from affected patients are recommended in estimating QALYs. To measure health-related quality of life, the SF-6D, EQ-5 and -15D are mostly recommended [17,19,25,27,30,35]. Using the same quality of life measurements, these measurements can be transferred between HTA agencies. However, even in this case, the transferability of results can be limited due to cultural differences [41]. Further research is therefore needed to investigate the differences of quality-of-life estimates between countries in order to assess whether QALYs can be transferred. Only willingness-to-pay is recommended for monetary valuation of outcomes. It can be presumed that for willingness-to-pay estimates, the differences between countries are even higher than for health-related quality of life [42]. Recommendations for sources of outcomes are conflicting. Some agencies prefer observational studies while others prefer experimental studies (above all, randomized controlled trials). This indicates the still ongoing, controversial discussion about whether the benefit of an intervention should be based on effectiveness or efficacy data and whether either can only be measured by one of the study types. On the one hand, it should be noted that because HTA evaluates mostly new products, observational data (e.g., registry data) are often not available. On the other hand, performing randomized controlled trials is expensive and time consuming and can delay the diffusion of a new HT. Where both types of data are found, the alleged gap between efficacy and effectiveness can be analyzed via sensitivity analysis to yield insight into the particular problem. This could be why some agencies do not restrict data in this manner and allow or require the use of both types of data [7,19,22,23,26,27]. Regardless of the study/data type recommended as a basis for analysis, performing a systematic review to obtain data is recommended by many agencies. Here, there is great potential for cooperation of HTA agencies to avoid duplicate work because a systematic literature search and selection of relevant effectiveness data is basically the same when the same HT is evaluated.

The divergences in recommended annual discount rates between HTA agencies are remarkable, which reflects the ongoing discussion about this issue [43,44]. The discussion primarily concerns differences between discount rates and benefit rates that can result in undesirable decisions. More agencies recommend using the same discount rate for costs as for benefits; fewer agencies recommend higher rates for costs than for benefits. Additionally, the recommended discount level varies for both costs and benefits. Although the discount rate is simple to adapt, it should be further harmonized because, depending on the time horizon of the analysis, it can have a strong influence on results and lead to unjustifiable differences in decisions between countries.

Recommendations on modeling are either not made by the agencies or are extremely vague. It can be presumed that this is because there are many different types of models and different problems that require different approaches. Perhaps, therefore, an a priori model specification is consciously avoided by the HTA agencies to prevent the use of suboptimal models for certain decisions; the detailed description for any potential model would be too extensive and always affected by uncertainty. The uncertainty in model choice and structure is also reflected in the fact that nine HTA agencies recommend testing different structural assumptions [7,19,22,25-27,30,32,35]. Specific recommendations are only made for decision trees and Markov-models, suggesting that these are the most established and accepted approaches for decision modeling [45]. Recommendations regarding the type of sensitivity analysis are partly contradictory. Some HTA agencies require exclusively either probabilistic or deterministic sensitivity analyses. There are also agencies that do not specify the type of sensitivity analysis, or allow both. It remains mostly unclear why some agencies prefer or accept only one of the two types of sensitivity analyses. The approaches to sensitivity analyses are based on different mathematical assumptions that can provide different insight on a decision. A restriction on a certain type of sensitivity analysis can be unrewarding. Thus, in the scientific literature no recommendation can be found in favor of using only one of the approaches [46] or using both [47] to test parameter uncertainty. An a priori specification of parameters that should be tested in a sensitivity analysis is not conducive because potentially all included results can influence sensitivity analysis [48]. Consequently, most agencies require only varying values for “relevant” parameters to prove the robustness of results. It also means that standardization is nearly impossible. For ranges, mostly confidence intervals are used. Surprisingly, incorporated differences that are presented in scientific literature are seldom requested. The recommended ranges for variation of discount rates are heterogeneous and lie often inside a wide range. This once again reflects a lack of consensus on this issue. Further recommendations on parameter variation are rare.

Equity aspects are given altogether little attention, which makes clear the often unbalanced view of HTA agencies between clinical effectiveness, cost-effectiveness and other aspects (social, ethical and legal). More efforts are needed to incorporate equity aspects into economic evaluations that reflect social values, especially given the background that reimbursement decisions depend on, and given the results of the economic evaluations, which can have different consequences depending on the patient group.

Similar to guidelines in scientific publications [49], the HTA agencies recommend mostly that results should be presented in disaggregated form and as incremental ratios. The disaggregated results support transparent reporting and incremental ratios simplify interpretation and conclusion synthesis. Both are important for transparent and trustworthy reimbursement decisions.

This study has some limitations. First, there is a language bias because we included only English and German manuals. Second, the response rate of the e-mail inquiry was only 28% and we contacted the HTA agencies only once for information, with no further attempts if a reply was not made. However it can be assumed that the investigation is fairly representative because there is no obvious reason that the HTA agencies make their recommendations not accessible on their webpages and the webpages of all HTA agencies were searched by two reviewers independently. Third, the comparison of methods might be partially inconsistent because of the different terminology used in the health economic literature. Fourth, only the members of the HTA umbrella organizations were considered.

## Conclusion

This is the first detailed analysis of methods used by international HTA agencies for economic evaluations. The number, detail, and content of recommendations vary strongly among HTA agencies. These findings are in accordance with prior research on HTA methods and procedures in general [50]. Differences in recommendations are often explainable by differences in the structure and regulation of health care systems and, therefore, different premises and goals. However, there are many conflicting recommendations for which the rationale is not obvious. It seems that some agencies place more value on the standardization of evaluations to ensure consistency, transparency and comprehensibility of evaluations. Others emphasize the flexibility of methods to allow choosing the best possible methods for each decision problem. HTA agencies in state-funded health care systems often have higher standardization, which is apparent in their more precise recommendations in the form of explicit guideline statements and stronger formulations.

Most of the recommendations are in accordance with described key principles of HTA [51,52]. These concern methods for assessing cost and benefits, the range of evidence and outcomes, the perspective of analysis and uncertainty of estimates. However, upon closer inspection, there are considerable unexplainable differences in methodological recommendations for economic evaluations. The results of HTAs always have generalizability and transferability restrictions for different populations and settings. Generalizability problems affect health outcomes and, more strongly, the costs of a HT [53]. However, difficulties in transferability should not yield a denial of standardization per se. First, the generalizability and transferability of study results is a general problem and thus also a problem if results are transferred within one country/jurisdiction. Additionally, there are process steps for the preparation of economic evaluations that are not at all, or only marginally, affected by transferability problems (e.g., literature search, model structure). More efforts should be made to harmonize the methods for independent preparation steps of economic evaluations. Furthermore, methods to enable a systematic identification of areas limiting generalizability and transferability need to be adapted in case a HTA is adapted for a different country. In existing umbrella organizations, such as EUnetHTA, such approaches could be developed (HTA-Core-Model) and the collaboration between HTA agencies promoted further. This is especially true because domains of potential for standardization of economic evaluations have been shown [40,54] and key principles for HTA are supported or still used by some HTA agencies [51]. Reliable benchmarking could support such a harmonization process [55]. Harmonization would facilitate the generalizability and transferability of results to different countries and could therefore help to avoid unnecessary duplicate work and expense [56]. Additionally, systematic reviews of health economic evaluations would become more informative if the results of HTAs were easier to generalize.

## Competing interests

The authors declare that they have no competing interests.

## Authors’ contributions

TM: development of study concept, literature search, selection of literature, data extraction, analysis of literature, draft of manuscript, final approval of the version submitted. EJ: literature search, review of manuscript, final approval of the version submitted. JM: literature search, review of manuscript, final approval of the version submitted. DP: quality assurance of data extraction, analysis of literature, review of manuscript, final approval of the version submitted.

## Pre-publication history

The pre-publication history for this paper can be accessed here:

http://www.biomedcentral.com/1472-6963/13/371/prepub

## Supplementary Material

Additional file 1: Table S1 Purpose, evaluated technologies and recommendations of HTA agencies. Click here for file
