# Supplementary material for: Methods of international health technology assessment agencies for economic evaluations- a comparative analysis
Source: BMC Health Serv Res. 2013 Sep 30;13:371. doi: 10.1186/1472-6963-13-371 (PMC3849629; doi:10.1186/1472-6963-13-371)
Supplement: Additional file 1: Table S1 — Purpose, evaluated technologies and recommendations of HTA agencies. [file 1472-6963-13-371-S1.doc]

**Additional file 1: Table S1 Purpose, evaluated technologies and recommendations of HTA agencies**

| **Agency** | **AHTApol 2009** | **CADTH 2006** | **CVZ 2006 / 2008** |
| --- | --- | --- | --- |
| **Item** |  |  |  |
| **Purpose** | Indicate the principles and basic methods of performing HTA to ensure high quality of analyses and reliable results. | Primary audience for the economic evaluations includes Canadian decision and policy makers who are responsible for the funding decisions regarding health technologies. | The CVZ uses these guidelines as an assessment framework for pharmacoeconomic evaluations which are part of a reimbursement file. This assessment framework is used to check whether the cost effectiveness of a drug for which reimbursement has been requested has been sufficiently substantiated. For reimbursement of drugs these guidelines are to be used. |
| **Evaluated technologies** | HTs not specified. | HTs not specified. | Intended for designing, conducting and reporting pharmacoeconomic research, but are applicable to economic evaluations in general within the field of health care. |
| **Types/choice of evaluation/outcomes** | CUA or CEA: standard. | CUA: should be used where meaningful HRQL differences between the intervention and alternatives have been demonstrated. | CUA: if improvement in the quality of life is an important effect. |
|  | CCA: standard. | CEA: should be used when a CUA is an inappropriate choice or as a secondary analysis when the use of an important patient outcome measure can be justified. | CEA: if improvement in the quality of life is not an important effect of the drug. |
|  | CMA: standard in case of no effectiveness differences. | CMA: where the evidence demonstrates that the important patient outcomes are essentially equivalent. | CMA: If the manufacturer does not expect the drug to have an added therapeutic value, nor that it will be mutually replaceable with different drugs. |
|  | CBA not recommended as basic method. | CBA: consequence of an intervention is difficult to value using QALYs, attribute of an intervention is difficult to value using any health outcome, process outcome are major factors in analyzing an intervention, secondary analysis. |  |
|  | Choice of one method does not exclude using another one as an additional analysis. | CCA is not preferred. |  |
| **Choice of alternatives** | Primary comparator: existing practice. | Comparator should be usual care. | Standard treatment, or if that does not exist, the usual treatment. |
|  | Furthermore recommended: | Recommended or appropriate care should be included. |  |
|  | most frequently used, cheapest, most efficient, compliant with standards and guidelines for clinical management. | In some instance, doing nothing or watchful waiting may be appropriate comparators. |  |
|  |  | Drugs: The comparators should include the lowest cost available alternative that is often used for the same indication. |  |
| **Time horizon** | Long enough to allow the assessment of differences between the results and costs. | The time horizon should be long enough to capture all the meaningful differences in costs and outcomes between the intervention and comparators. | Must be such that it enables valid and reliable statements to be made regarding the effects and costs of the treatments being compared. |
|  | Same for cost measurement and health. | Analysts are encouraged to consider a lifetime time horizon as a default, particularly for chronic conditions. |  |
|  |  | In some cases, multiple time horizons might be appropriate for the extrapolated data. |  |
| **Perspective** | Primary: entity financing health care services. | Publicly funded health care system. | Societal perspective. |
|  | Secondary (author considers it significant in the process of specifying the recommendations): social perspective. | Costs associated with adopting a wider perspective should be reported separately where it is likely that they have an impact on the results of the analysis. |  |
| **Costs** | Only the costs corresponding to consumable resources used during the application. | Publicly funded health care system: direct costs to publicly funded health care system, direct costs to patients and their families, time costs to patients and their families. | Societal perspective: direct and the indirect costs inside and outside the healthcare system. |
|  | Social perspective: direct medical costs, direct non-medical costs, indirect costs. | Societal perspective: direct costs to publicly funded health care system, direct costs to patients and their families, time costs to patients and their families, productivity costs. | All are included, irrespective of who actually bears the costs or receives the benefits. |
| **Measurement/ sources of resource use / costs** | Unit cost. | Natural units. | When indirect costs outside the health care system are involved, these need to be calculated using the friction cost method. |
|  | Primary data within a properly designed research, or by collecting secondary data from existing databases. | Resource use data can be obtained from several sources, including RCTs, administrative and accounting data, clinical practice guidelines, expert opinion, and modeling exercises. | Possible data sources are the patients themselves, their care-providers or existing registries. |
|  | Micro-costing method is better suited to the interventions and events occurring at the present moment. | Detailed micro-costing can produce more precise estimates, although the time and expense of collecting such data need to be considered. |  |
|  | Gross-costing is acceptable, when the implementation of the more accurate micro costing method shall have no significant impact on the analysis results. |  |  |
| **Valuating of resource use** | Unit costs: list of standard costs, formerly published research, local scales of charges, direct calculation. | Valued at their opportunity cost. | Unit cost; official invoice prices (standard prices), current market prices. |
|  |  | Market prices, administrative fees, direct measurement, and calculation of shadow prices No consensus regarding the best method of valuation. | Where possible making use of standard prices and taxe-prices and not tariffs. |
| **Measurement / valuation of outcomes** | CUA: Indirect methods for preferences measurement, use of direct tools of preference measurement is not excluded if needed for the subject. | HRQL: preference-based measures should be used | Descriptive quality-of-life questionnaires cannot be used as a measurement. |
|  |  | QALYs: analysts are encouraged to use indirect measurement instruments. |  |
|  |  | It is preferred that analysts measuring preferences directly use a representative sample of the general public. |  |
|  |  | CBA: WTP. |  |
| **Sources for outcomes** | Results of effectiveness obtained from observational studies are better than experimental results assessed in a SR. | SR of the available evidence on the efficacy and effectiveness of the intervention. | Data on the comparative treatment can be based on retrospective data sources (RCTs, patient files) or on prospective research (observational studies or patient registries). |
| **Discounting per year** | Cost: 5%. | Cost and outcomes: 5%. | Costs: 4%. |
|  | Health care results: 3.5%. |  | Effects: 1.5%. |
| **Modeling** | If data in the model are extrapolated over time horizon of the primary trials, the following scenarios should be analyzed: optimistic, pessimistic and neutral. | SRs and meta-analyses can produce high quality data for model parameters. | Preferably based on ‘peer-reviewed’ publications. |
|  | Half-cycle correction should be implemented to adjust time-dependent assessment. |  |  |
|  | Input data: a SR of the relevant literature should be carried out to obtain the crucial input data for the model. |  |  |
| **Analysis of sensitivity /uncertainty** | DSA and PSA are acceptable. | DSA (minimum requirement): Analysis for all model inputs to determine the impact on the results, assess the full range of plausible values for each parameter. | With respect to the uncertainty of deterministic variables, univariate SA need to be conducted. |
|  | SA of the crucial parameters and a justification of the analyzed range of parameter variability, first of all those input data for which the scatter measures and estimation uncertainty are the highest. | PSA (encouraged to more appropriately assessing parameter uncertainty): Monte Carlo simulation, quantification of the contribution of each parameter to decision uncertainty. | In relation to the uncertainty of stochastic variables, PSA need to be carried. |
|  | Parameter variability should be determined on the basis of a review of publications, experts’ opinions or confidence intervals around the average value. | Discounting: 0% and 3% (cost and outcomes) discount rate. | In the case of an empiric cost-effectiveness analysis based on primary, patient-related data, the uncertainty of costs, effects and the cost-effectiveness relationship need to be reflected with distribution measures. In addition to statistical uncertainty, within empirical studies there remains also the need of univariate SA. |
|  | At least a simple one-way and multi-way SA. | Model uncertainty should be assessed through a DSA and model validation methods, with separate (probabilistic) results shown for each alternative analysis. |  |
|  | Discounting: 5% for costs and health care results, 0% for costs and health care results, 0% for health care results and 5% for costs. |  |  |
| **Equity aspects** | NR | Identify the equity-relevant characteristics of the subgroups that may benefit from, or be adversely affected by, the intervention. | NR |
|  |  | Analysts are encouraged to provide information on the distributional impact and cost effectiveness of the intervention for those subgroups predetermined to be relevant for equity purposes. |  |
|  |  | Use equal equity weights for all outcomes. |  |
| **Presentation of results** | NR | Present the analysis in disaggregated detail first, showing total, undiscounted costs and outcomes separately for the intervention and each comparator. | NR |
|  |  | Introduce aggregations, incremental results, and value judgments as late as possible. |  |
|  |  | Report final results as ICERs, based on incremental differences of expected costs and expected outcomes of the alternatives. |  |
|  |  | Follow standard decision rules for estimating ICERs, including the exclusion of dominated alternatives. To aid understanding, analysts are encouraged to present the results of the analysis in graphical or visual form, in addition to tabular presentation. |  |

| **Agency** | **DACEHTA 2007** | **GOEG 2012** | **HIQA 2010** |
| --- | --- | --- | --- |
| **Item** |  |  |  |
| **Purpose** | The handbook is aimed at anyone who takes part in planning and implementing of HTA projects and/or seeks HTA to be carried out, i.e. health professionals, political and administrative decision-makers, interest groups, researchers and others who want to adopt an HTA approach, including teachers and students. | Methods described in the handbook should be applied for all publically funded HTA projects. | Intended to inform economic evaluations conducted by, or on behalf of the HIQA, the National Centre for Pharmacoeconomics, the Department of Health and Children and the Health Service Executive (HSE), to include health technology suppliers preparing applications for reimbursement. |
| **Evaluated products** | HTs not specified. | HTs not specified. | HTs not specified. |
| **Types/choice of evaluation/outcomes** | NR | CUA, CEA, CBA, CMA (no specification what should be done or what is preferred). | CUA: preferred evaluation type. |
|  |  |  | CEA: in exceptional circumstances with the outcomes expressed in terms of life-years gained may be used when a CUA is an unsuitable choice, secondary analysis when the use of an important patient outcome (other than a QALY) can be justified. |
|  |  |  | CMA: if empirical justification using robust scientific evidence is provided to support the claim that there is no meaningful difference in terms of important patient outcomes. |
| **Choice of alternatives** | The assessed technology should be compared with alternative technologies, primarily with the best established practice. | Should be comprehensible and transparent. | Preferred comparator for ‘routine care (technologies most widely used in clinical practice). |
|  |  |  | Comparators are not limited to specific interventions, but may include alternative treatment sequences or alternative rules for starting and stopping therapy. |
|  |  |  | It is feasible that there will be more than one appropriate comparator technology because of variations in routine practice. |
| **Time horizon** | NR | Influence of intervention on resource use and health effects must be proven by data for the chosen time horizon. | Sufficient duration to capture any meaningful differences in the future costs and outcomes likely to accrue to the competing technologies. |
|  |  |  | A lifetime horizon is usually considered appropriate as the majority of technologies have costs and outcomes that impact over a patient’s lifetime. |
| **Perspective** | Generally recommended that the economic analysis is based on the broadest perspective possible (societal perspective). | Society, public health care system, social insurance, health insurance funds, hospitals, physicians, patients, depending on research question. | Publicly-funded health and social care system. |
|  | If an economic analysis is to be used for societal prioritization, it must of course have a societal perspective, as the prioritization otherwise risks becoming suboptimal. |  | If the inclusion of a wider societal perspective is expected to impact on the results of the analysis significantly, this may be presented as a secondary analysis in addition. |
| **Costs** | Health care sector: direct cost for hospitals and health care sector. | Payer perspective: all direct medical and, if relevant, indirect medical costs (e.g., transport). | Publicly-funded health and social care system: direct costs. |
|  | Hospital direct cost for hospitals. | Societal perspective: all direct medical and, if relevant, indirect medical costs (e.g., transport) and productivity losses due to morbidity. |  |
|  | Societal perspective: direct cost (hospital, health care sector, other sector, patient family), production loss/gain, future costs. |  |  |
|  | Intangible costs are not included. |  |  |
| **Measurement/ sources of resource use / costs** | Physical units. | According to the type of resource use (e.g., pharmaceuticals, care, rehabilitation). | Physical units and unit costs. |
|  | Recommend the use of prospective designs. | Micro-costing is preferred. | Data for resource use and costs should be identified systematically. |
|  | The aim for an HTA is to be based on the existing literature, it will often be found to be impossible to collect prospective economic data, and deterministic data must therefore be used instead. | Cost data should come from the specific context. | Resource use data can be obtained from the literature or by primary data collection. Sources include RCTs, meta-analysis, clinical practice guidelines, local administration and accounting data, and expert opinion. |
| **Valuating of resource use** | Ideally, the price of resource consumption must correspond to the opportunity cost. | NR. | Value added price taxes (VAT) should be excluded. |
|  | As opportunity costs are difficult to measure in practice, however, it is often necessary to use rates. |  | For non-drugs, the public list price should be used. |
|  |  |  | Discounts should also be accounted for. |
|  |  |  | In general, the public list price paid for a drug should be used. |
|  |  |  | In the absence of a published list price, the price submitted by a manufacturer for a technology may be used. |
|  |  |  | Cost should reflect that of the product, formulation and pack size that gives the lowest cost. |
|  |  |  | Drug cost estimates should reflect mandatory rebates from pharmaceutical manufacturers and importers. |
|  |  |  | Labor (pay) should be calculated using consolidated salary scales. |
|  |  |  | Associated non-pay costs should be estimated in accordance with the methods outlined in the Regulatory Impact Analysis guidelines issued by the Department of the Taoiseach. |
|  |  |  | Costs for the most recent calendar year should be used with retrospective input costs inflated using the Consumer for health Transfer payments. |
| **Measurement / valuation of outcomes** | QALYs: generally measured using EQ-5D or 15D. | QALYs: preference-based measures should be used. Use of standardized indirect methods is preferred. | QALYs: indirect preference-based methods such as the EQ-5D or SF-6D are recommended to measure utilities. |
|  | CBA: WTP methods should not be used alone. | If determined by direct methods, standard-gamble and time-trade should be used. |  |
|  |  | Surrogates must have been proven. |  |
| **Sources for outcomes** | Source for data on the effectiveness of health technologies is the medical literature. | RCTs or SRs/meta-analysis of RCTs. For estimation of effectiveness (long-term data) other study designs could be used. | SR of all high-caliber, relevant data. Where available, evidence RCTs should be used to quantify efficacy. Meta-analysis may be used to synthesize outcome data provided. |
|  | CEA: In selecting data on effect, internal and external validity must therefore be weighed up. |  | Experimental, quasi-experimental and non-experimental or observational data may be submitted to supplement the available RCTs. |
| **Discounting per year** | No values specified. | Cost and outcomes: 3%. | Costs and benefits: 4%. |
| **Modeling** | NR | Decision analysis. | For state transition the cycle length should be sufficiently short to ensure that multiple changes in disease, treatment decisions or costs do not occur within a single cycle. |
|  |  | Model type (Markov-model, decision-tree) should not be determined a priori. |  |
|  |  | Based on all relevant evidence. |  |
|  |  | Meta-analysis of data should be used (if possible). |  |
| **Analysis of sensitivity / uncertainty** | Independently for costs and effects. | For all relevant parameters based on confidence intervals. | Key inputs should be subjected. |
|  | Confidence intervals are estimated for the combined incremental ICER either by bootstrapping, when patient-specific data are available, or by Monte Carlo simulation, when data are deterministic. | Recommended one way DSA. | Analyses for the range of plausible scenarios. |
|  | Discounting: discount rate should be varied in the SA. | The consideration of the whole uncertainty targeting a PSA must be performed. | One-way SA should be conducted to identify the key model inputs / assumptions. |
|  | Model structure:where appropriate, be tested with setting-up of alternative models. | The bias of epidemiological studies should be regarded. | Multivariate SA should be used for key model inputs. |
|  |  | Discounting: 0%, 5%, 10%. | Assumptions about the model structure should be clearly stated and justified and their impact on cost-effectiveness explored though a series of plausible scenario analyses.PSA in the form of a Monte Carlo simulation should be used to assess parameter uncertainty. |
|  |  | A verification of the model should be performed. | To illustrate the impact of costs on the results, costs should be varied by +/− 20% in one-way SA. |
|  |  | If possible, the model should be eternally validated. | Discount rate should be varied from 0% to 6% in the univariate SA. |
|  |  | If possible, different structural assumptions should be tested by using a SA. | SA in the form of threshold analysis may also be used when the baseline value of a parameter is unknown. |
| **Equity aspects** | NR | Relevant groups (e.g., age disease stadium) should be considered separately. | Additional QALYs gained should be assumed to be of equal value, regardless of any considerations for specific characteristics of the population. |
| **Presentation of results** | When cost-effect ratios, including ICERs, are calculated and compared for the health technologies considered, and conclusions are reached about their cost-effectiveness, the results must be incorporated. | Detailed description of results. | All results should be presented in both their disaggregated and aggregated form. |
|  |  |  | Expected mean costs, total costs and QALYs should be documented for the comparator technologies with ICERs calculated, as appropriate. |
|  |  |  | Uncertainty should be presented graphically and in tabular form to facilitate interpretation. |
|  |  |  | The probability that a technology is cost effective at a range of threshold levels should also be presented. |

| **Agency** | **INFARMED 1998** | **IQWIG 2008/2009/2011** | **KCE 2008** |
| --- | --- | --- | --- |
| **Item** |  |  |  |
| **Purpose** | For the assessment of an application for co-payment of a drug, the applicant should submit an economic assessment study conducted according to the rules defined by order of the Ministry of Health. | Support to ascertain an upper limit for reimbursement of pharmaceuticals. | For pharmacoeconomic evaluations submitted in the context of a reimbursement request for pharmaceutical products for which pharmacoeconomic assessment is either compulsory or useful or a revision file 1.5 to 3 years after the initial reimbursement decision. |
|  | A guide of good practice that was sufficiently general to be used in any institutional context and could, with a few changes, be applied to the assessment of all health technologies. | Description of principles as bases for the evaluation of efficiency of an intervention compared with the efficiency of an existing intervention for a certain indication. | The guidelines are built around a reference case that defines the recommended methodology for each component of the economic evaluation. |
| **Evaluated products** | Drugs. | Pharmaceuticals. | Drugs. |
| **Types/choice of evaluation/outcomes** | CUA: whenever possible to make it possible to compare the results of the studies of different pathologies. | Not specified (see row: outcomes). | CUA: if the treatment has an impact on health related quality of life that is significant to the patient or if there are multiple patient-relevant clinical outcome parameters expressed in different units. |
|  | CEA: if consequences associated with all the alternatives are not the same. |  | CEA: if improving life expectancy is the main objective of the treatment and also the most important outcome. |
|  | CMA: consequences associated with all the alternatives are the same. |  | CBA: are not accepted as reference case. |
|  | CBA: to make it possible to compare the results of the studies of different pathologies if CUA is not possible. |  |  |
| **Choice of alternatives** | Reference alternative should be that of current practice. | All relevant pharmaceutical interventions for the indication (cost-effectiveness frontier). | Most relevant alternative treatment for the proposed indication. If this treatment cannot be identified, the recommended treatment according to the Belgian clinical guidelines should be used as a comparator. |
|  | If the most common treatment is not the one recognized as the most efficacious, or is not the cheapest of the efficacious treatments, these treatments should also be used as terms of comparison. |  | In some cases, multiple treatments will have to be included as comparator. |
|  |  |  | Off-label use of products should not be used as a comparator. |
| **Time horizon** | The length of the study should coincide with the duration of the treatment and its consequences. | Time horizon should be chosen according to the course of the disease to allow evaluation of all costs. | Depends on the natural history of the disease. |
|  |  | Chronic conditions: life time. | For chronic diseases and acute diseases with long-term sequelae, a lifetime horizon should be applied. |
| **Perspective** | Perspective should be that of society. | Perspective of social insured (not social insurance). | Cost: health care payer. |
|  |  | Perspective of the society in case of relevance. | Outcomes: societal perspective. |
| **Costs** | Society: direct costs of providing health care, costs of social services and other sectors related to health care and the costs borne by patients and their families. The only indirect costs included should be those of an employee’s lost productivity. | Perspective of social insured: direct medical costs (including co-payments), direct non-medical costs if relevant. | Health care payer: direct health care costs (payments out of the government’s health care budget as well as patients’ co-payments). |
|  |  | Societal perspective: indirect costs. |  |
| **Measurement/ sources of resource use / costs** | Physical units. | Determination of the resource utilization must be based on current data and quality assured. | Measurement of resource use should be done by means of observations or derived from literature. |
|  | Information on the use of resources should be based on clinical practice in the country. If this is not possible, it is necessary to use foreign data, they should be validated by local health care providers. | Productivity loss: human capital approach. | Use of expert panels for resource use measurement is subject to specific conditions. |
| **Valuating of resource use** | Units of measurement should reflect the opportunity cost of these resources. | Opportunity costs of the society. | Principle of the cost analysis is that costs are valued at opportunity costs. |
|  |  | Societal perspective: for health resources it is recommended to use resource prices (e.g., personal) | Opportunity costs will be approximated by market prices or some kind of mechanism used for the reimbursement of procedures (e.g. per diem price). |
|  |  | Medical resources should be valued at market prices. | Where generic pharmaceutical products exist, the reference price for these products should be used in the pharmacoeconomic evaluation. |
|  |  | For pharmaceuticals estimation, marked shares or adapted market prices is recommended. | Valuation of resource use by means of simple currency conversion of values found in literature or in studies from other countries is not acceptable. |
|  |  |  | All costs should be expressed in values for the current year (years should be inflated using the appropriate Health Index figures). |
| **Measurement / valuation of outcomes** | CUA: Whenever possible, it is advisable to present results based on generic measurements and specific instruments at the same time. | Outcomes: Especially mortality, morbidity and validated surrogates as outcomes. | CUA/QALYs: Health-related quality of life weights should be based on empirical data, obtained with a descriptive system for health status for which corresponding preference values exist from the general public. |
|  | CEA: End points we consider should, as far as possible, be those related to the impact of treatments on the duration of life. |  | Quality of life weights should be derived with a generic instrument. |
|  | CBA: WTP should be assessed using the contingent valuation method. |  | The use of Belgian preference values is preferred. |
|  |  |  | Scenarios with disease-specific measures for health-related quality of life can be presented as complementary analyses. |
|  |  |  | CEA: expressed in terms of life years gained for chronic conditions and acute conditions with long term sequelae or a relevant short term outcome for acute conditions with no long term consequences. |
| **Sources for outcomes** | Preference will be given to the results obtained in clinical trials with validated methodologies and relevance. | Data should be searched systematically. | Life expectancy should be estimated based on age-specific life tables. |
|  | Effect should be assessed in terms of effectiveness whenever possible. If this information is not available, the use of efficacy data will be accepted. | Effectiveness: RCTs (non-randomized and observational studies only in justified exceptions). | Should always be based to some extent on data from RCTs or non-interventional studies comparing the study product and a relevant comparator. Economic evaluations based on active control studies are preferred. |
|  |  | Expert opinions should be avoided. |  |
| **Discounting per year** | Cost and consequences: 5%. | Cost and consequences: 3%. | Costs: 3%. |
|  |  |  | Benefits: 1.5%. |
| **Modeling** | NR. | Modeling technique should not be determined a priori. | If modeling is needed because clinical trials provide insufficient information for the economic evaluation, the number of assumptions not based on clinical evidence should be reduced to a minimum. |
| **Analysis of sensitivity / uncertainty** | SA of the key parameters with values that are subject to uncertainty. | It is recommended not to substitute important univariate DSA by multivariate PSA but to use PSA in addition. | PSA should be performed on all uncertain parameters |
|  | Analysis should be conducted considering the confidence intervals for each estimate. | PSA: correlations between parameters must be incorporated in Monte-Carlo simulation. | For composite measures, such as total costs, the different components with their respective distributions should be included in the sensitivity analysis. |
|  | Parameters should be justified in detail on the basis of empirical evidence or of logic. | Regarding cost of leisure time loss is allowed. | In addition to PSA, a scenario or univariable SA could be performed on modeling parameters that are decisive for the cost-effectiveness ratio. |
|  | Discounting: If the consequences are not valued in monetary terms, the SA should include the zero rates. | Discount rate should be varied from 0% to 5%. | The applicant is free to present additional univariable sensitivity or scenario analyses. |
|  |  | Structural SA should be performed to determine the influence of variation of structural assumptions. | Methodological uncertainty arising from the applied discount rate or the extrapolation method used in models should be tested using scenario analysis. This is comparable to one-way SA, where only one parameter is changed. |
| **Equity aspects** | NR | NR | NR |
| **Presentation of results** | The presentation of economic assessment studies should comply with a form identifying all relevant aspects needed to understand the analysis of the studies. | Results should be presented as ranges instead of point estimates. | Results should be presented in a tabular form. The table should contain the discounted costs, outcomes, incremental costs and incremental outcomes in a disaggregated form and separately for the study intervention and the comparator. Incremental cost-effectiveness ratios should be presented if the treatment is not dominant. |
|  |  |  | For the presentation of cost data, the expected total costs of each alternative should be presented as well as the average incremental cost, together with its confidence interval. Unit costs (in Euros) and quantities of resources used should be reported separately. |
|  |  |  | Uncertainty analysis: present cost-effectiveness or cost-utility plane, cost-effectiveness acceptability curve and/or incremental net benefit diagram, present confidence interval around the incremental cost-effectiveness ratio. |

| **Agency** | **MAS 2012 (last modification of webpage)** | **MSAC 2005** | **NICE 2008/2009** |
| --- | --- | --- | --- |
| **Item** |  |  |  |
| **Purpose** | Approach that manufacturers of pharmaceuticals can use to satisfy the information needs of Ontario Public Drug Programs. | When MSAC assesses a new service, it is required to consider the comparative cost and cost-effectiveness of the service as well as the effectiveness and safety. Therefore, applications and assessment reports should include an economic evaluation. | Describes key principles of appraisal methodology and is a guide for all organizations considering submitting evidence to the technology appraisal program of the Institute |
|  | Does not reflect new criteria or regulations. | Description of an appropriate approach to constructing an economic analysis to inform a decision. | Details what the Institute considers to be appropriate methods for assembling and synthesizing evidence on the technology being appraised in order to estimate its clinical and cost effectiveness. |
| **Evaluated products** | Drugs. | HT not specified. | Pharmaceuticals, medical devices, diagnostic techniques, surgical procedures, other therapeutic technologies, health promotion activities. |
| **Types/choice of evaluation/outcomes** | Evaluations that consider both the comparative costs associated with use of pharmaceutical products and the comparative clinical effects measured either in pure clinical units (effectiveness), or in health preferences (utilities), or clinical outcomes [e.g., quality adjusted life years (QALYs)], or dollars (benefits). | CUA, CEA, CBA, CMA, CCA (choice is based on a classification algorithm that regards: effectiveness and safety/harm). | CE (specifically CUA) analysis is the preferred form of economic evaluation. |
|  | It is expected that efficacy, and perhaps effectiveness, will have already been demonstrated by RCTs with low risks of false positive and false negative errors. | CBA: not preferred. |  |
|  |  | Various types of analyses should not be considered mutually exclusive. |  |
| **Choice of alternatives** | Comparisons should be made with the least expensive currently available strategy. | Currently available service that is most likely to be replaced by the new service. | Therapies routinely used in the NHS, including technologies regarded as current best practice. |
|  | Also be made with the most commonly used alternative product. |  |  |
| **Time horizon** | The analysis should delineate the time horizon on which estimates can be based from currently available high quality empirical data or from modeled data based on extrapolations. | Appropriate time horizon for follow-up will relate to the natural history of the disease, the treatment pattern and the time period over which outcomes from the service or main comparator could be expected to occur. | The time horizon should be sufficient to reflect important cost and benefit differences between the technologies being compared. |
| **Perspective** | Societal perspective. | Societal perspective. | Outcomes: all direct health effects, whether for patients or, when relevant, other people (principally carers). |
|  |  |  | Costs: National Health System and personal social services. |
| **Costs** | Societal perspective: direct costs, including those borne outside the healthcare system, and indirect costs such as lost wages. | Health care resources, resources consumed in sectors other than the health care sector, patient and family resources, production changes. | National Health System and personal social services. |
|  |  |  | Other government bodies (substantial impact on the costs). |
|  |  |  | Costs borne by patients may be included when they are reimbursed by National Health System. |
| **Measurement/ sources of resource use / costs** | Unit costs. | Physical units. | Unit costs. |
|  | Direct measurement of resources used. | Analyses should be based on the best evidence available. | Resource use and cost data should be identified systematically. |
|  | Some investigators will have to use a sampling technique to estimate the quantity of individual services delivered under competing strategies. Others can use published information from other clinical trials. |  | National data based on healthcare resource groups should be considered National Health System and Personal social services. |
| **Valuating of resource use** | Unit prices for the resources need to be estimated in Canada. | Opportunity cost. | Unit costs should be routinely obtained from national list prices. |
|  |  | Medical services: schedule prices. | Resources should be valued using the prices relevant to National Health System and Personal social services. |
|  |  | Hospital services: DRGs. | VAT should be excluded from all economic evaluations. |
|  |  | Diagnostic: schedule prices. |  |
|  |  | Pharmaceuticals: schedule prices. |  |
|  |  | Allied health care services: resource manual. |  |
|  |  | Where more than one unit cost for a service is, the lowest should be assumed. |  |
| **Measurement / valuation of outcomes** | QALYs: only instruments measuring the utility for general health status are acceptable. | QALYs: preferred method of measuring is multi-attribute utility instrument. | QALYs: HRQL should be reported directly from patients and/or carers, based on public preferences using a choice-based method, EQ-5D is the preferred measure. |
|  | Utility: estimated in a sample of subjects, with the disorder of interest. | Acceptable multi-attribute utility instruments are the Health Utilities Index, the EQ5D, the SF-6D or the Assessment of Quality of Life instrument. |  |
|  | Monetary outcome measures: WTP |  |  |
| **Sources for outcomes** | A comprehensive search method is used to locate all relevant studies, the variations in findings of the studies are analyzed, and the results of primary studies are combined in an appropriate manner. | SR | SR |
|  |  |  | Head-to-head RCTs provide the most valid evidence of relative treatment effect. However, such evidence may not always be available and may not be sufficient to quantify baseline health effects. Therefore, data from non-randomized studies may be required to supplement RCT data. |
| **Discounting per year** | NR | Costs and benefits: 5%. | Costs and benefits: 3.5%. |
| **Modeling** | NR | Decision analytic methods | NR |
| **Analysis of sensitivity / uncertainty** | QALYs: extensive SA to demonstrate that the conclusions of the analyses are not sensitive to the type of measurement chosen. | SA should be presented which examine the effect of varying assumptions in the valuation of outcomes. | The implications of different estimates of key parameters must be reflected in SA. |
|  | Difference between efficacy and effectiveness must be considered. | Where the lowest price is not assumed in the base case analysis, SA for lowest price. | Parameter precision: PSA is preferred. |
|  |  | SA examining the impact of discounting should be performed. | Evidence about the extent of correlation between individual parameters should be carefully considered and reflected. |
|  |  | SA should be conducted to explore the impact of uncertainty in the quantification of resources. | The impact of structural uncertainty on estimates of cost effectiveness should be explored by separate analyses of a representative range of plausible scenarios. |
|  |  | SA substituting the National Public Sector Cost Weight for the National Private Sector Cost Weight should be provided. |  |
|  |  | Non-health related benefits should not be included in the base-case analysis but may be included in a SA. |  |
| **Equity aspects** | NR | NR | An additional QALY has the same weight regardless of the other characteristics of the individuals receiving the health benefit. |
| **Presentation of results** | Reporting the CEA should be provided. | The results of each step of the economic evaluation should be presented, firstly in disaggregated form, then in increasingly aggregated form. | The expected value of each component of cost and expected total costs should be presented; expected QALYs for each option compared in the analysis should also be detailed in terms of their main contributing components. ICERs should be calculated as appropriate. |
|  |  | The appropriately aggregated and discounted results should be presented separately for outcomes and resources and separately for the proposed service and ist main comparator. |  |
|  |  | For cost-effectiveness and cost-utility analyses, the application or assessment report should present the incremental cost of achieving each additional unit of outcome when the proposed service is substituted for the main comparator. |  |
|  |  | Uncertainty: where there are multiple outcomes, the application or assessment report should consider providing a matrix with the effects of variables on various outcomes that differ across the two arms. The application or assessment report should present the results of multiway SA on variables shown to be important drivers of results in the one-way analyses. Results should be presented in tabular form and as graphs. Results should also be presented diagrammatically in the form of a scatter plot on the cost-effectiveness plane and, if desired, an acceptability curve. |  |

| **Institution** | **PBAC 2008** | **PHARMAC 2009/2012** |  |
| --- | --- | --- | --- |
| **Item** |  |  |  |
| **Purpose** | These Guidelines for Preparing Submissions to the PBAC (referred to in this document as the ‘PBAC Guidelines’) provide practical information for the pharmaceutical industry for making a submission to PBAC. | The PHARMAC Board has nine decision criteria to weigh up when making funding decisions, of which cost-effectiveness is only one. Analysis describes the approach we take when doing cost-utility analysis. It is also a guide for pharmaceutical suppliers when undertaking their own economic analyses to support new funding applications. |  |
|  | Although the guidelines have been written for the pharmaceutical industry, they are also intended to help PBAC assess submissions and provide information to other interested stakeholders, including clinical and patient groups, and the general community. |  |  |
|  | Economic evaluation is one factor to be considered when making choices between competing therapeutic modalities. |  |  |
| **Evaluated products** | Drugs. | Drugs. |  |
| **Types/choice of evaluation/outcomes** | CUA: preferred when there is a claim of incremental life years gained in the economic evaluation in order to assess the impact of quality adjusting that survival gain or relevant direct RCT report results using a multi-attribute utility instrument. | CUA: most analyses undertaken by PHARMAC. |  |
|  | CEA: if the proposed drug is demonstrated to offer more of a given health outcome than its main comparator. |  |  |
|  | CMA: if the proposed drug has been shown to be non-inferior (equivalent) to the main comparator. |  |  |
|  | CCA: it can be presented if the proposed drug is demonstrated to have a different profile of effects that are not adequately captured by a single outcome measure; there might be trade-offs between the two drugs in terms of the directions of the changes in effectiveness and safety. |  |  |
|  | CBA: is not preferred (supplementary only). |  |  |
|  | Various types of analyses should not be considered mutually exclusive. |  |  |
| **Choice of alternatives** | The main comparator is defined as the therapy that prescribers would most replace with the proposed drug in practice. | Treatment that most prescribers or clinicians would replace and the treatment prescribed to the largest number of patients. |  |
|  |  | Costs and savings to other (non-healthcare) government departments should be discussed in the report if significant. |  |
| **Time horizon** | Appropriate time horizon for follow-up relates to the natural history of the medical condition, the treatment patterns, and an estimation of the time period(s) over which outcomes from the two therapies would be expected to occur. | In the majority of CUAs a lifetime horizon should be used. |  |
| **Perspective** | Seeks to take the perspective of society. | Perspective of the funder. |  |
| **Costs** | Direct health care resources. | CUA: pharmaceutical costs, health sector costs (and cost savings) and direct patient health care costs. |  |
|  | All contributions to the costs of health care resources, including those paid for by patients, governments, health insurance agencies and any other part of society, should be considered for inclusion in the economic evaluation. | Indirect costs should not be included. |  |
|  | Supplementary an economic evaluation incorporating changes in non-health care resources. |  |  |
| **Measurement/ sources of resource use / costs** | Natural units. | Pharmaceuticals: dose used in the key clinical trials. |  |
|  | The pattern of provision of resources may be measured prospectively in the course of a clinical study, by retrospective review of relevant records, by administration of a questionnaire or survey, or through the use of diaries. |  |  |
|  | Do not include as consequences in the economic evaluation other unrelated medical conditions that, in the fullness of time, are likely to afflict patients who live longer as a result of effective treatment that they receive now. |  |  |
| **Valuating of resource use** | Opportunity cost. | Pharmaceuticals: schedule prices, take into account any rebate from the pharmaceutical supplier. |  |
|  | Price per unit. | Cost of co-administered and comparator treatments: schedule prices. |  |
|  | Pharmaceuticals: schedule prices. | Pharmacy fees & mark ups: dispensing fees should be included. |  |
|  | Medical services: schedule prices. | Hospital Inpatient Costs: DRGs. |  |
|  | Hospital services: DRGs. | Outpatient hospital visits: specialist consultation cost or same-day DRG costs |  |
|  | Diagnostic: schedule prices. |  |  |
|  | Allied health care services: resource manual. |  |  |
|  | Production changes: the friction method is theoretically preferable to the human capital method. |  |  |
| **Measurement / valuation of outcomes** | Generally be based on the outcome measure that most closely and validly estimates the final health outcome from a patient perspective. | QALYs: only the HRQL of the patient being treated should be included, value-judgment weightings not be included, EQ-5D Tariff 2 should be referred |  |
|  | QALYs: generally preferred method of measuring QALYs is by the |  |  |
|  | Where utility weights were not elicited via a multi-attribute utility instrument in the direct RCTs, this might form a basis for valuing these effects in a manner that reflects the preferences of the general population. |  |  |
|  | Acceptable multi-attribute utility instruments are the Health Utilities Index, the EQ5D, the SF-6D or the Assessment of Quality of Life instrument. |  |  |
| **Sources for outcomes** | PBAC has a strong preference for clinical and economic evaluations that are based on direct RCTs. | Clinical effect: All appropriate levels of evidence should be identified; however well-conducted RCTs and meta-analyses are the preferred data sources |  |
|  | However, PBAC recognizes that direct RCTs are not always available. If this is the case, alternatives might be (in order of priority): an indirect comparison across two or more sets of RCTs or non-randomized studies. |  |  |
|  | Literature review: the search should involve at least four approaches: |  |  |
|  | (a) a search of the published literature |  |  |
|  | (b) a search of registers of RCTs |  |  |
|  | (c) an examination of the dossier seeking marketing approval submitted to the Therapeutic Goods Administration, supplemented by checks with the sponsor’s head office and subsidiaries of the company (and any other original sponsor or co-licensed companies) for any further RCTs (which may be unpublished) |  |  |
|  | (d) manual checking of reference lists of all relevant articles that are obtained by other means. |  |  |
|  | Repeated application of a valid, reliable and responsive multi-attribute utility instrument questionnaire to participants in RCTs, together with the application of an appropriate scoring algorithm. |  |  |
| **Discounting per year** | Costs and outcomes: 5%. | Costs and benefits: 3.5%. |  |
| **Modeling** | NR | Decision trees and Markov models. |  |
|  |  | Markov models: half cycle adjustment applied. |  |
| **Analysis of sensitivity / uncertainty** | Different discount rates (including a zero discount rate on non-monetary outcomes alone and on both costs and outcomes) should be tested. | SA should include univariate (simple) analysis and multivariate analysis. When undertaking detailed analysis, PSA may be necessary. Any uncertainty in the analysis should be fully tested. |  |
|  | If a decision to exclude or include one or more studies is likely to be controversial, it is usually wiser to also present a SA examining whether the decision makes a difference to the conclusions from the overall clinical evaluation. | Parameters to consider include those with the greatest level of uncertainty, and those with the greatest influence on model outcomes. |  |
|  | Equity: see line equity aspects. | Range over which parameters should be varied in the SA should be based on the available scientific literature, expert opinions, or a scale that is regarded as plausible. |  |
|  | Financial estimates. | Discounting cost and benefits: 0% and 5%. |  |
|  | Estimates of drug use for the PBS context. | It is recommended that structural uncertainty be formally examined in SA. When testing the model, we consider that extreme SA should be used to ensure that the model generates logical results. |  |
|  | Each time an assumption is required in the absence of data. |  |  |
|  | If there is a risk of substantial usage beyond the intended population and circumstances of use defined in the requested restriction, examine the sensitivity of the results to the assumption of usage within these intentions. |  |  |
|  | If a cost-utility analysis is presented, also present the results of the economic evaluation with the utility in all health states set to one to generate the incremental cost per extra life year gained. |  |  |
|  | Examine assumptions concerning the structure of the modelled economic evaluation that are uncertain to assess their importance by the extent to which they affect the results of the evaluation. |  |  |
| **Equity aspects** | In the rare cases in which such underlying assumptions might be important enough to influence a particular PBAC decision, a description of how the issue affects consideration of the cost**-**effectiveness of the drug and preferably an examination of its impact in a SA should be sufficient. | NR |  |
|  | If the intention of the restriction is to limit usage to the population for which the proposed drug is most cost-effective, these SA should examine the extent to which the incremental cost-effectiveness ratio would become less favorable with increasing usage beyond the restriction. |  |  |
| **Presentation of results** | Present the cost per patient per course if the proposed drug is for acute or self-limited therapy, or the cost per patient per year if the proposed drug is for chronic or continuing therapy. | Disaggregation of costs, savings, lifeexpectancy and quality of life gains/losses. Discounted incremental QALYs/$ (point estimate and range) and corresponding cost/QALY results (point estimate and range. |  |
|  | Present the remaining results of the economic evaluation first in a disaggregated form, then in increasingly aggregated forms. | Uncertainty: report using graphs, tables and/or elasticities. Include a full interpretation of the results. |  |
|  | Present the appropriately aggregated and discounted results separately for outcomes and costs, and separately for the proposed drug and its main comparator. |  |  |
|  | Present separate estimates of the incremental cost and the incremental effectiveness of substituting the proposed drug for the main comparator. |  |  |
|  | For cost-effectiveness and cost-utility analyses, present the incremental ICER as the incremental cost of achieving each extra unit of outcome with the proposed drug substituted for the main comparator (the base case of the economic evaluation). |  |  |

CUA: cost utility analysis; CEA: cost effectiveness analysis; CCA; cost consequences analysis; CMA: cost minimization analysis; CBA: cost benefit analysis; HRQAL: health related quality of life; HT: health technology; ICERs: Incremental cost-effectiveness ratios NR: no recommendations; QALYs: Quality adjusted life years; RCTs: randomized controlled trials; WTP: willingness to pay; SR: systematic review; SA: sensitivity analysis; DSA: deterministic sensitivity analysis; PSA: probabilistic sensitivity analysis; ICER: incremental cost effectiveness ratio; VAT: value added price taxes; DRG: diagnosis related groups
